# Informational masking in the modulation domaina)

**DOI:** 10.1121/10.0005038

**Published:** 2021-05-27

**Authors:** Christopher Conroy, Gerald Kidd

**Affiliations:** Department of Speech, Language & Hearing Sciences and Hearing Research Center, Boston University, 635 Commonwealth Avenue, Boston, Massachusetts 02215, USA

## Abstract

Uncertainty regarding the frequency spectrum of a masker can have an adverse effect on the ability to focus selective attention on a target frequency channel, yielding informational masking (IM). This study sought to determine if uncertainty regarding the modulation spectrum of a masker can have an analogous adverse effect on the ability to focus selective attention on a target modulation channel, yielding IM in the modulation domain, or “modulation IM.” A single-interval, two-alternative forced-choice (yes-no) procedure was used. The task was to detect 32-Hz target sinusoidal amplitude modulation (SAM) imposed on a broadband-noise carrier in the presence of masker SAM imposed on the same carrier. Six maskers, spanning the range from 8 to 128 Hz in half-octave steps, were tested, excluding those that fell within a two-octave protected zone surrounding the target. Psychometric functions (*d′*-vs-target modulation depth) were measured for each masker under two conditions: a fixed (low-uncertainty/low-IM) condition, in which the masker was the same on all trials within a block, and a random (high-uncertainty/high-IM) condition, in which it varied randomly from presentation-to-presentation. Thresholds and slopes extracted from the psychometric functions differed markedly between the conditions. These results are consistent with the idea that IM occurs in the modulation domain.

## I. INTRODUCTION

There is considerable psychophysical evidence to support the conceptual model of amplitude-modulation processing as mediated by an array of channels tuned to modulation frequency (e.g., Houtgast, 1989; Bacon and Grantham, 1989; Dau *et al.*, 1997; Dau *et al.*, 1999; Ewert and Dau, 2000; Ewert *et al.*, 2002; Ewert and Dau, 2004; Sęk and Moore, 2003; Wojtczak and Viemeister, 2005; Moore *et al.*, 2009; Wojtczak *et al.*, 2011; Wojtczak, 2011; Sęk *et al.*, 2015; Füllgrabe *et al.*, 2021). The function of this putative array of channels is to decompose a stimulus comprising complex modulations into its constituent modulation frequencies, thereby enabling internal access to different regions of its amplitude-modulation spectrum.

Psychophysical modulation-masking experiments illustrate this notion. In a typical modulation masking experiment, the observer's task is to detect the presence of target modulation—for example, sinusoidal amplitude modulation (SAM)—imposed on a carrier in the presence of masker modulation imposed on the same carrier. A modulation masking pattern is obtained by measuring target-SAM detection thresholds as a function of the modulation frequency of the masker SAM, with the modulation depth of the masker held fixed (e.g., Strickland and Viemeister, 1996; Ewert *et al.*, 2002; Wojtczak, 2011; Sęk *et al.*, 2015). What is typically found is that the modulation depth of the target at detection threshold is highest (poorest) for maskers that are close to the target in terms of modulation frequency, and lowest for those that are remote, with a gradual transition in between. In other words, a peaked masking pattern emerges, with a peak centered at or near the modulation frequency of the target.

Assuming detection *via* modulation channels, the interpretation of this pattern of results is straightforward: the peak in the masking pattern (i.e., modulation masking) reflects competition between the target SAM and the masker SAM within a single, attended-to modulation channel (cf. Dau *et al.*, 1997; Dau *et al.*, 1999; Ewert and Dau, 2000; Ewert *et al.*, 2002; Ewert and Dau, 2004; Wojtczak, 2011). When the target and the masker are close in terms of modulation frequency, large amounts of masking occur because the masker strongly stimulates the attended-to channel, obscuring weak targets and raising detection thresholds in turn. When the target and the masker are remote in terms of modulation frequency, minimal masking occurs because the masker only weakly stimulates the attended-to channel—strongly driving some flanking channel instead—and thus can easily be ignored by the observer when making a detection decision. In the frequency domain, masking that results from competition between a target and a masker within a single frequency channel is often referred to as energetic masking (EM) (cf. Durlach *et al.*, 2003a). Thus, by way of analogy, modulation masking such as that described above can be interpreted as EM in the modulation domain inasmuch as it reflects competition between the target SAM and the masker SAM within a single modulation channel (cf. Durlach *et al.*, 2003a; Sheft and Yost, 2007).

An interpretation of modulation masking in terms of EM has intuitive appeal and accords well with the results of many modulation-masking experiments (see references above). Yet, in the frequency domain, it has long been known that there are certain masked detection conditions under which EM alone is insufficient to explain performance. For example, when masker-frequency uncertainty is created in a masked pure-tone detection experiment by randomly varying the frequency spectrum of the masker from presentation-to-presentation (e.g., Neff and Green, 1987), large amounts of masking can occur that cannot be attributed solely to EM (for a review of many such experiments, see Kidd *et al.*, 2008). Instead, the preponderance of masking in such cases likely arises because uncertainty regarding the frequency spectrum of the masker has an adverse effect on the ability to focus selective attention on the target frequency channel and ignore the uninformative flanking channel(s) on each trial (e.g., Lutfi, 1993; Neff *et al.*, 1993; Allen and Wightman, 1995; Oh and Lutfi, 1998; Wright and Saberi, 1999; Richards *et al.*, 2002; Tang and Richards, 2003; Lutfi *et al.*, 2003; Durlach *et al.*, 2005). In this article, we report the results of an experiment designed to answer the following question: Does uncertainty regarding the modulation spectrum of a masker in a modulation masking experiment have an analogous adverse effect on the ability to focus selective attention on a target modulation channel?

The term informational masking (IM) is often used to refer to interference in detection performance that cannot be attributed to EM (cf. Durlach *et al.*, 2003a). For a particular masking effect to qualify as IM, however, it is often stipulated that it must be the result of uncertainty arising from random or unexpected variations in a masker along one or more acoustic dimensions (e.g., Watson and Kelly, 1981; Neff and Green, 1987; Watson, 1987; Neff and Callaghan, 1988; Lutfi, 1990; Oh and Lutfi, 1998; Lutfi *et al.*, 2003), although other factors (e.g., target-masker similarity; Kidd *et al.*, 1994; Neff, 1995; Kidd *et al.*, 2002; Durlach *et al.*, 2003b; Lee and Richards, 2011) may contribute as well. Thus, when masker-frequency uncertainty is created in a masked pure-tone detection experiment by randomly varying the frequency spectrum of the masker from presentation to presentation, the masking that results is considered to be due to IM. In this article, we refer to it as IM in the frequency domain, or “frequency IM,” to denote the following: (1) frequency is the dimension along which the detection-relevant and irrelevant flanking channels are tuned; (2) the uncertainty that produces the masking is the result of random or unexpected variations in the frequency spectrum of the masker; and (3) the hypothesized consequence of the uncertainty is an inability to focus selective attention on the target frequency channel. Thus, swapping “frequency” for “modulation” in each of these criteria and considering modulation masking as EM in the modulation domain, the question posed in the preceding paragraph can be reformulated as follows: Is there such a thing as IM in the modulation domain, or “modulation IM”?

We are not unique in posing this question. Durlach *et al.* (2003a) noted that while the vast majority of past research on IM has focused on the frequency domain, it could easily occur in other, “nonfrequency domains” (p. 2985) as well, insofar as channel-based processing is a factor therein. Indeed, they suggested the modulation domain as a possibility in a parenthetical remark (see Durlach *et al.*, 2003a, p. 2985), but never reported the results of such an experiment, or elaborated further. Sheft and Yost (2007) conceptualized modulation-detection-interference (MDI)—a phenomenon in which masker modulations in one carrier-frequency region interfere with the detection of target modulations in another—as an IM-like effect. The basis for their conceptualization was that the observed pattern of MDI could not be explained in terms of competition between the target and masker modulations within modulation-tuned channels; that is, by EM in the modulation domain. Although their findings and interpretation do satisfy many of the criteria typically applied to IM and thus could be considered as modulation IM, they differ from the view proposed here in two important respects. First, our conceptualization of modulation IM depends on the central role of uncertainty and considers masker-modulation-frequency uncertainty resulting from random or unexpected variations in the modulation spectrum of a masker to be fundamental to modulation IM. Masker-modulation-frequency uncertainty was not an element of the design and interpretation of the experiments reported by Sheft and Yost (2007). Second, we emphasize a theoretical account of IM in which the hypothesized consequence of uncertainty is an inability to focus selective attention on a target channel tuned in the domain in which the uncertainty exists. Although the empirical results reported by Sheft and Yost are consistent with such a failure, this was not the mechanism they invoked to explain their findings.

In the experiment reported here, target-SAM detection performance was measured in a modulation masking experiment very much like the “typical” modulation masking experiment described above only modified to include, in the key condition, masker-modulation-frequency uncertainty. Masker-modulation-frequency uncertainty was created in a manner analogous to the way in which masker-frequency uncertainty is often created in masked pure-tone detection experiments concerned with frequency IM, namely, by randomly varying the modulation frequency of the masker SAM from presentation to presentation. Any adverse effect of uncertainty in this context was taken as evidence of modulation IM. Our working hypothesis was that, if evident, modulation IM would reflect an adverse effect of uncertainty on the ability to focus selective attention on the target modulation channel.

## II. METHODS

### A. Observers

Eight observers (four males; 20–31 years; mean = 23 years) participated. All observers had pure-tone air-conduction thresholds within the normal range at octave frequencies from 250 to 8000 Hz. One observer was an author (C.C.); another was a researcher in our lab. Recruitment and use of human subjects protocols were approved by the Boston University Charles River Campus Institutional Review Board.

### B. Apparatus

Stimuli were generated using matlab (MathWorks, Inc., Natick, MA), routed through a 24-bit sound card (RME HDSP 9632, Haimhausen, Germany), and presented monaurally to the left ear *via* a pair of headphones (Sennheiser HD280 pro, Wedemark, Germany). Observers performed the task individually while seated in a double-walled, sound-treated booth (Industrial Acoustics Company, North Aurora, IL) equipped with a computer monitor, a keyboard, and a mouse.

### C. Stimuli and procedures

The details of the experimental design followed closely those described by Durlach *et al.* (2005) in their study of frequency IM. A single-interval, two-alternative forced-choice (yes-no) procedure was used. The task was to detect target SAM (the target) of a fixed and known modulation frequency imposed on a broadband noise carrier with a 0.5 *a priori* probability on each trial. Simultaneous masker SAM (the masker) was imposed on the same noise carrier with a 1.0 *a priori* probability on each trial. The carrier was 500 ms and was different on each trial. The masker and, when present, the target, were applied to the entire 500-ms duration. On masker-alone trials, the equation that described the waveform was

$$[1+m_{m}\;\text{sin}(2\pi f_{m}t)]n\left(t\right),$$ (1)

where *m_m_* was the modulation depth of the masker, *f_m_* was the modulation frequency of the masker in Hz, and *n*(*t*) was the noise carrier. On target+masker trials, the equation that described the waveform was

$$[1+m_{m}\;\text{sin}(2\pi f_{m}t)+m_{t}\;\text{sin}(2\pi f_{t}t)]n\left(t\right),$$ (2)

where *m_t_* was the modulation depth of the target and *f_t_* was the modulation frequency of the target in Hz.

The modulation frequency of the target was 32 Hz. The modulation depth of the masker was 0.5, or –6 dB in terms of 20log(*m_m_*). Six maskers were tested, spanning the range from 8 to 128 Hz in half-octave steps. They were 8, 11, 16, 64, 91, and 128 Hz. Maskers that fell within a two-octave protected zone surrounding the target were excluded in order to minimize the effects of modulation masking (i.e., EM in the modulation domain) so that any effects of modulation IM could be more readily identified.

On each trial, the waveform described by either Eqs. (1) or (2) was bandpass filtered between 80 and 8000 Hz using an 8th order Butterworth bandpass filter, ramped using 50-ms cosine-squared onset-offset ramps, and scaled to an overall level of 50 dB sound pressure level (SPL) before presentation to the observer during a 500-ms observation interval. The observation interval was preceded by a 500-ms warning light (displayed on the in-booth monitor) and followed by a response period of unlimited duration. The observer's response (“target present,” yes-no) was registered *via* mouse click on a labeled button displayed on the in-booth monitor. Once registered, no opportunity for corrections was provided and correct-answer feedback was displayed immediately on the monitor for 500 ms before the next trial commenced or the block of trials terminated.

Target-SAM detection performance was measured in two stimulus conditions: a fixed condition, denoted *F*, in which the masker was the same on all trials within a block, and a random condition, denoted *R*, in which the masker was selected at random on each trial from the set of six. The use of the same masker across trials in the *F* condition was assumed to preclude masker-modulation-frequency uncertainty (or at least that masker-modulation-frequency uncertainty was at a minimum) and thus the *F* condition was considered the low-uncertainty/low-IM reference condition. The presentation-to-presentation randomization of the masker in the *R* condition was intended to result in (or to increase the amount of) masker-modulation-frequency uncertainty (relative to the *F* condition) and thus the *R* condition was considered the high-uncertainty/high-IM comparison condition. It is worth reiterating here that when we say “the masker” and refer to its randomization we are referring to the imposed masker modulator. The noise carrier, which was unique on each trial, had inherent fluctuations of its own that also could have produced modulation masking (e.g., Dau *et al.*, 1999) and/or uncertainty (cf. Spiegel and Green, 1982). Target-SAM detection performance was evaluated for each masker in each stimulus condition as a function of target modulation depth and quantified *via* the standard detection-theory index of sensitivity *d′*. Three target modulation depths spaced 5 dB apart were tested for each masker, with the specific depths chosen to ensure that a wide range of performance was obtained for each masker. For a given masker, the same three depths were tested in both the *F* and *R* conditions.

Prior to beginning data collection, each observer completed six adaptive tracks of unmasked target modulation detection to gain familiarity with the target. A two-interval, two-alternative forced-choice procedure was used and correct-answer feedback was provided on all trials. The same stimuli were used during the familiarization period as were used during the experimental session save for the fact that a masker was never present.

During the experimental session, trials were completed in blocks of 50 and *F* and *R* blocks were completed in alternation, always beginning with an *F* block. In the *F* condition, the target depth was the same on all trials within a block. The three target depths that were tested for each masker were rank-ordered (highest to lowest) and, following Durlach *et al.* (2005), were tested in decreasing order across blocks to minimize any uncertainty associated with variability in target depth that might have arisen had we adapted on target depth or used the method of constant stimuli. A block of trials was completed for each masker at each rank-ordered value before proceeding to the next rank-ordered value for any other masker. The order (different for each observer) in which the six maskers were tested at each rank-ordered value was randomized across blocks. Note that this randomization procedure applied to the *F* condition only; in the *R* condition, the masker was selected at random on each trial. Moreover, in the *R* condition, the target modulation depth was fixed within a block according to its rank-ordered value (as opposed to its absolute value, as in the *F* condition) and the three rank-ordered values were tested in decreasing order across blocks.

The data collection procedure described above yielded a 2 × 2 stimulus-response matrix (i.e., number of hits, misses, false alarms, and correct rejections) based on 50 trials for each combination of target depth, masker, and stimulus condition. It was completed in a single experimental session lasting roughly 1.5 h. After this first experimental session was completed, it was repeated (on a different day, preceded by two additional unmasked target modulation detection blocks for the purposes of refamiliarization with the target) to arrive at the final data set for each observer, i.e., a 2 × 2 stimulus-response matrix based on 100 trials for each combination of target depth, masker, and stimulus condition.

### D. Analysis conditions

Only two stimulus conditions were tested, *F* and *R*. Following Durlach *et al.* (2005), however, the final data set for each observer in each stimulus condition was subjected to two separate analyses, yielding *d′* values for two pairs of analysis conditions. For the two “pooled” analysis conditions, *F_p_* and *R_p_*, all 2 × 2 stimulus-response matrices associated with a particular target depth and stimulus condition were pooled across maskers (i.e., the numerical values in each cell were summed across maskers) and *d′_p_* was calculated on the basis of these pooled matrices. In the second pair of analysis conditions, detection performance was evaluated at the individual masker level. For these two “sorted” analysis conditions, *F_s_* and *R_s_*, the 2 × 2 stimulus-response matrices associated with each target depth were sorted by masker and stimulus condition and *d′_s_* was calculated on the basis of these sorted matrices.

Regardless of the analysis condition, each 2 × 2 stimulus-response matrix was converted to a matrix of response probabilities (i.e., probability of a hit, miss, false alarm, and correction rejection), extreme probabilities were corrected using the “1/(2 N)” rule (Hautus, 1995), and *d′* was computed *via* the standard equation for yes-no tasks (see, e.g., Macmillan and Creelman, 2005, p. 8); that is,

$$d^{\prime }=z\left(P_{H}\right)-z\left(P_{FA}\right),$$ (3)

where *z*(*P_H_*) was the *z*-score of the probability of a hit and *z*(*P_FA_*) was the *z*-score of the probability of a false alarm.

## III. RESULTS

**FIG. 1.**
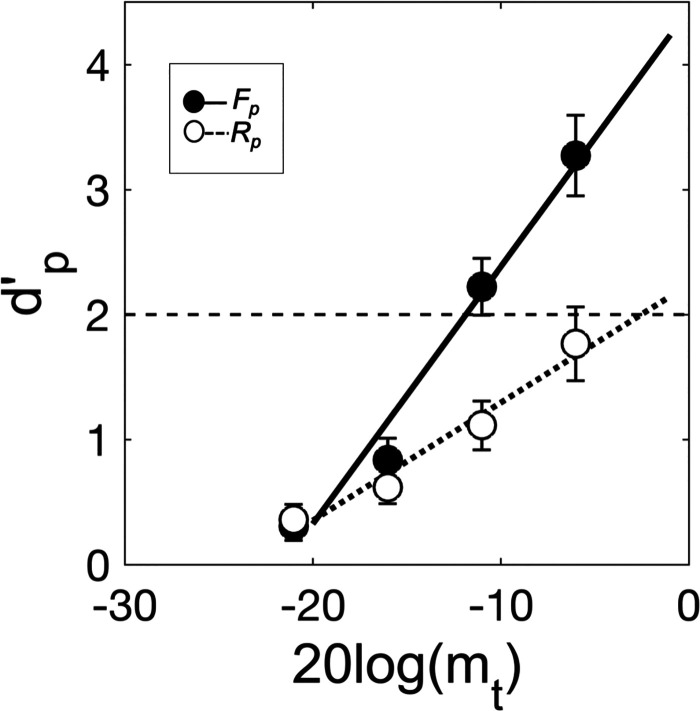
Group-mean detectability (*d′_p_*) plotted as a function of target modulation depth in dB for each of the two pooled analysis conditions: the low-uncertainty/low-IM *F_p_* condition (filled symbols) and the high-uncertainty/high-IM *R_p_* condition (open symbols). Error bars give ±1 SE of the mean. Also shown are group-mean psychometric functions for the *F_p_* (solid line) and *R_p_* (dotted line) conditions (see the text for details). The horizontal dashed line marks *d′_p=2_*, or threshold-level performance.

**FIG. 2.**
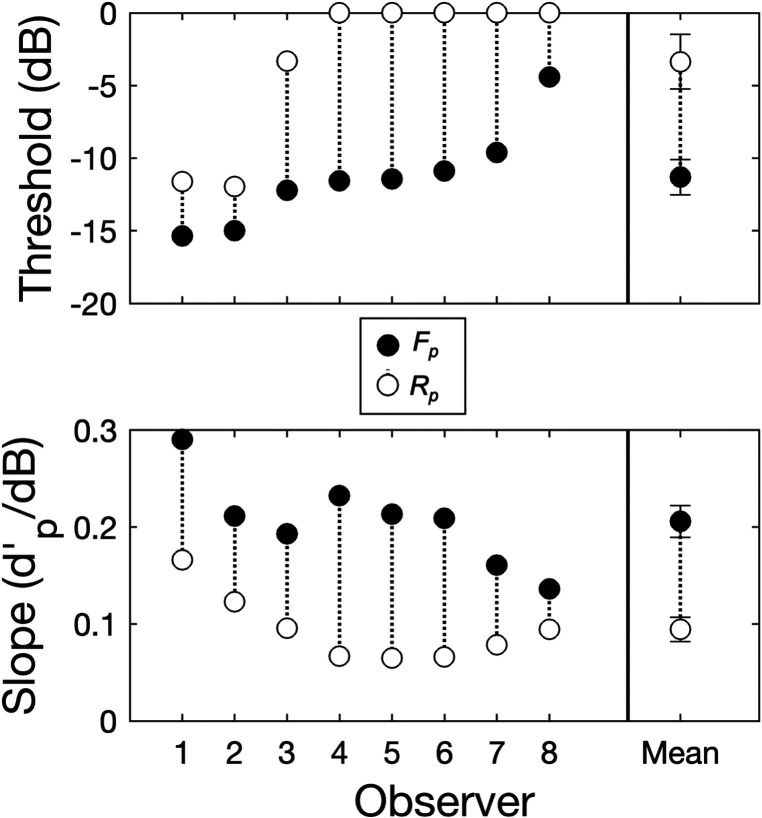
Individual and group-mean thresholds (top panel) and psychometric function slopes (bottom panel) in each of the two pooled analysis conditions, *F_p_* and *R_p_*. Error bars give ± 1 SE of the mean.

Figure 1 shows group-mean *d′_p_* plotted as a function of target modulation depth in dB for each of the two pooled analysis conditions (cf. Sec. II D), *F_p_* (filled symbols) and *R_p_* (open symbols). Error bars give ± 1 standard error (SE) of the mean. Also shown are group-mean psychometric functions for the same two conditions, *F_p_* (solid line) and *R_p_* (dotted line). The group-mean psychometric function for each condition was obtained by averaging the parameters (slope and *y*-intercept) of the individual psychometric functions, fit separately to the *d′_p_* data for each observer in each condition. The individual psychometric functions were computed as linear least squares fits to the *d′_p_* data on the coordinates of *d′_p_*-vs-target modulation depth in dB. Following Durlach *et al.* (2005), threshold-level performance was defined as the target modulation depth in dB at which the fitted psychometric function crossed *d′_p=2_*; following Sęk *et al.* (2015), if the fitted function failed to cross *d′_p=2_* by a target modulation depth of 0 dB, the threshold was set to 0 dB to acknowledge the constraint of overmodulation (referred to as “the 0-dB maximum threshold rule” in what follows). Individual and group-mean thresholds (top panel) and psychometric function slopes (bottom panel) are plotted in Fig. 2. Note that in Fig. 2, the two observers from our lab, author C.C. and another researcher, are Observers 1 and 2, respectively.

Turning first to the thresholds: thresholds were higher (poorer) in the *R_p_* condition than in the *F_p_* condition for all observers, indicating an adverse effect of masker-modulation-frequency uncertainty on target SAM detection. This, we argue, is modulation IM. Individual *F_p_* thresholds ranged from −15 to −4 dB and the group-mean *F_p_* threshold was –11 dB (SE = 1 dB). *R_p_* thresholds ranged from −12 to 0 dB, with a group mean of −3 dB (SE = 2 dB). Because modulation masking (i.e., EM in the modulation domain) was equivalent in both the low-IM *F_p_* and high-IM *R_p_* conditions, the difference between each observer's *F_p_* and *R_p_* threshold (*R_p_* −*F_p_*) provides an index of modulation IM analogous to that which is typically used to index frequency IM (cf. Lutfi, 1990; Durlach *et al.*, 2003a). In general, a positive threshold difference indicates the presence of modulation IM and larger threshold differences indicate greater susceptibility, whereas smaller threshold differences indicate greater resilience (with the caveat that the 0-dB maximum threshold rule put a cap on threshold differences in some cases; cf. Fig. 2). Threshold differences ranged from 3 to 12 dB. The group-mean threshold difference was 8 dB (SE = 1 dB). A one-tailed t-test confirmed that the mean threshold difference was significantly greater than zero [*t*(7) = 6.20, *p* < 0.001], suggesting that modulation IM was a significant factor in the *R_p_* condition.

Turning now to the *d′_p_* psychometric functions: consistent with the results of numerous past studies of frequency IM (e.g., Neff and Callaghan, 1988; Kidd *et al.*, 1994; Wright and Saberi, 1999; Lutfi *et al.*, 2003; Tang and Richards, 2003; Kidd *et al.*, 2003; Durlach *et al.*, 2005), psychometric functions were shallower in the high-IM *R_p_* condition than in the low-IM *F_p_* condition, again for all observers. The slopes of the *F_p_* functions ranged from 0.14 to 0.29 *d′_p_*/dB and the group-mean *F_p_* slope was 0.21 *d′_p_*/dB. The slopes of the *R_p_* functions ranged from 0.06 to 0.17 *d′_p_*/dB and the group-mean *R_p_* slope was 0.10 *d′_p_*/dB. Unlike the threshold-difference metric, the ratio of psychometric function slopes (*F_p_*/*R_p_*) for each observer provides an index of modulation IM (cf. Durlach *et al.*, 2005) uncontaminated by the 0-dB maximum threshold rule. A slope ratio ≠ 1 indicates an effect of modulation IM. Slope ratios ranged from 1.44 to 3.47 and the group-mean slope ratio was 2.36 (SE = 0.29). A two-tailed t-test confirmed that slope ratios were significantly greater than one [*t*(7) = 4.76, *p* < 0.01], suggesting that that modulation IM yielded a significant decrease in the slope of the psychometric function for target SAM detection.

**FIG. 3.**
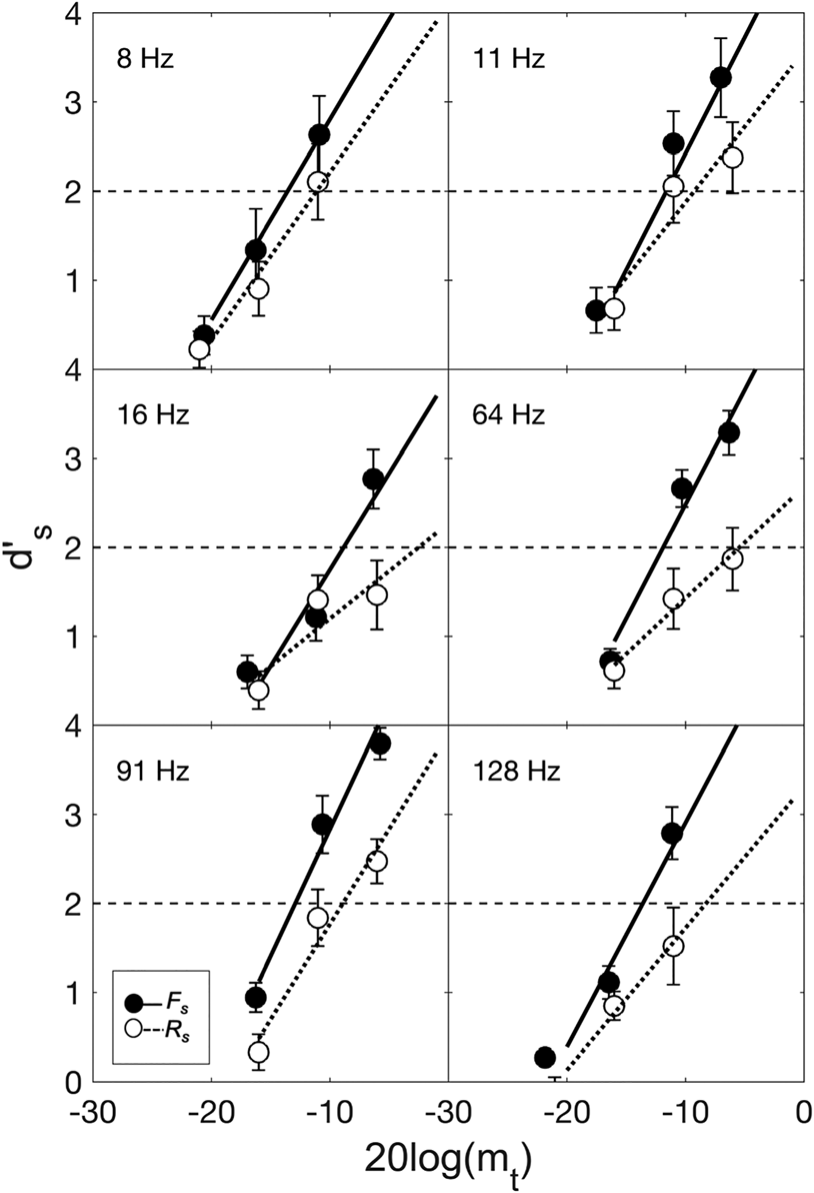
Group mean *d′_s_* plotted as a function of target modulation depth in dB for each masker (panels, masker modulation frequency inset) in each of the two sorted analysis conditions, *F_s_* (filled symbols) and *R_s_* (open symbols). Error bars show *±*1 SE of the mean. Note that the symbols have been jittered along the abscissa slightly to improve clarity. Also shown are group mean psychometric functions for each masker in the *F_s_* (solid lines) and *R_s_* (dotted lines) conditions. The horizontal dashed line in each panel marks *d′_p_* = 2, or threshold-level performance.

The pooled analysis presented above was used to examine the effect of uncertainty at the masker ensemble level; the sorted analysis presented below, on the other hand, was used to examine the effect of uncertainty at the individual masker level (cf. Sec. II D). As with the *d′_p_* data, the *d′_s_* data for each observer, each masker, and each condition were fit with a straight-line psychometric function on the coordinates of *d′_s_*-vs-target modulation depth in dB and thresholds and slopes were extracted from the fitted psychometric functions as described above; group-mean psychometric functions were obtained for each masker in each condition, again, by averaging the parameters (slope and *y*-intercept) of the individual observer fits. Figure 3 shows group-mean *d′_s_* plotted as a function of target modulation depth in dB as well as the group-mean psychometric function for each masker (different panels, masker modulation frequency inset) in each of the two sorted analysis conditions, *F_s_* (filled symbols, solid lines) and *R_s_* (open symbols, dotted lines). Error bars give ± 1 SE of the mean.

**FIG. 4.**
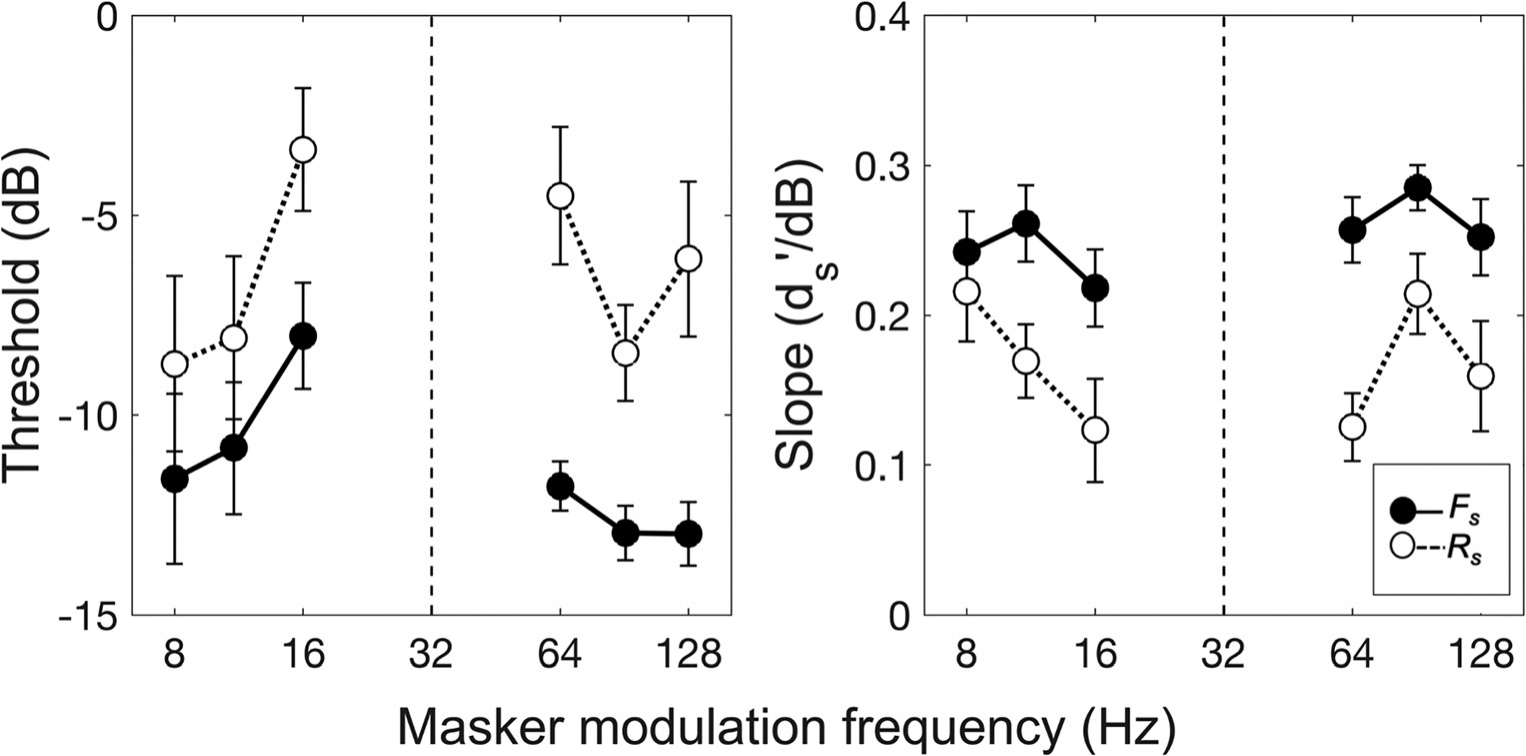
Group-mean thresholds (left panel) and psychometric function slopes (right panel) for each masker plotted as a function of the modulation frequency of the masker in each of the two sorted analysis conditions, *F_s_* (filled symbols, solid lines) and *R_s_* (open symbols, dotted lines). Error bars give *±*1 SE of the mean. The vertical dashed line marks 32 Hz, the modulation frequency of the target.

Consistent with the *d′_p_* results above, the *d′_s_* results in Fig. 3 show that masker-modulation-frequency uncertainty had a generally adverse effect on target-SAM detection performance across maskers, yielding both a positive threshold difference (*R_s_* −*F_s_*) and a slope ratio (*F_s_*/*R_s_*) > 1 in all cases. This is illustrated more clearly in Fig. 4, which shows group-mean thresholds (left panel) and psychometric function slopes (right panel) for each masker plotted as a function of the modulation frequency of the masker in both the *F_s_* (filled symbols, solid lines) and *R_s_* (open symbols, dotted lines) conditions. Error bars give *±* 1 SE of the mean. The vertical displacement of the *F_s_* and *R_s_* data in each panel of Fig. 4 illustrates the effect of uncertainty on thresholds (left panel) and slopes (right panel): *R_s_* symbols above *F_s_* symbols in the left panel of Fig. 4 indicate positive threshold differences, whereas *R_s_* symbols below *F_s_* symbols in the right panel of Fig. 4 indicate slope ratios >1.

A two-way repeated-measures analysis of variance (ANOVA) performed on the thresholds revealed that both the main effects of uncertainty [*F_s_* vs *R_s_*, *F*(1,7) = 31.70, *p* < 0.001] and masker modulation frequency [six modulation frequencies, *F*(2.37,16.61) = 3.86, *p* < 0.05, Greenhouse-Geisser correction] were significant.^1^ Notably, the interaction term (uncertainty × masker modulation frequency) approached, but did not reach, statistical significance at the *p* < 0.05 level [*F*(5,35) = 2.12, *p* = 0.09], indicating that threshold differences were comparable across masker modulation frequencies. The lack of a statistically significant interaction comes with a caveat, however, in that it may reflect, in part, the 0-dB maximum threshold rule, which limited the dynamic range available to measure threshold differences for some maskers, and, in particular, for the two maskers immediately flanking the target.^2^ Indeed, visual inspection of the group-mean psychometric functions shown in Fig. 3 suggests that threshold differences tended to be relatively large for the two maskers immediately flanking the target and that, without an “artificial” cap of 0 dB, threshold differences may have been larger still.

A second ANOVA performed on the psychometric function slopes indicated that, as expected, the main effect of uncertainty was significant [*F_s_* vs *R_s_*, *F*(1,7) = 29.79, *p* < 0.001]. Neither the main effect of masker modulation frequency [six modulation frequencies, *F*(3.09, 21.61) = 2.08, *p* > 0.05, Greenhouse-Geisser correction] nor the interaction between uncertainty and masker modulation frequency [*F*(5,35) = 1.02, *p* > 0.05] were significant, however, indicating that while masker-modulation-frequency uncertainty may have yielded flatter psychometric functions overall, the degree of flattening was similar across maskers.

## IV. DISCUSSION

The purpose of this study was to answer a relatively simple question: Is there such a thing as IM in the modulation domain, or, what we have called, modulation IM? In short, the results suggest: Yes. Analyzed at the masker ensemble level (pooled analysis conditions), target-SAM detection thresholds were poorer, and psychometric functions were flatter, when masker-modulation-frequency uncertainty was high than when it was low (Figs. 1 and 2). Analyzed at the individual masker level (sorted analysis conditions), masker-modulation-frequency uncertainty had a generally adverse effect on thresholds across maskers (Figs. 3 and 4), yielding shallower psychometric functions as well (Figs. 3 and 4). Because modulation masking (i.e., EM in the modulation domain) was equated across both the *F* and *R* conditions through the use of a protected zone and the same set of six maskers, we argue that these findings reflect the effects of modulation IM.

As noted in Sec. I, our working hypothesis was that, if evident, modulation IM would reflect an adverse effect of masker-modulation-frequency uncertainty on the ability to focus selective attention on a target modulation channel. The pattern of thresholds and slopes summarized above is generally consistent with this hypothesis. For example, if it is assumed that, in the *F* condition, observers were able to focus their attention on the target modulation channel but that in the *R* condition were either distracted or confused by the masker on some trials and therefore attended to the “wrong” (i.e., nontarget) modulation channel instead, an increase in thresholds and decrease in psychometric function slopes in the high-IM *R* condition relative to the low-IM *F* condition would be expected (cf. Allen and Wightman, 1995; see also Hübner, 1993; Green, 1995). Insofar as all maskers were equally distracting/confusing, this account would predict a similar effect of uncertainty on both thresholds and slopes across maskers (i.e., comparable threshold differences and slope ratios across maskers) and therefore would be consistent both with the results on *d′_p_* (Figs. 1 and 2) and on *d′_s_* (Figs. 3 and 4). It also suggests an important role for target-masker confusions in producing modulation IM (cf. Sheft and Yost, 2007), very much like how target-masker confusions can be an important factor in producing frequency IM (e.g., Kidd *et al.*, 1994; Neff, 1995; Kidd *et al.*, 2002; Durlach *et al.*, 2003b; Lee and Richards, 2011).

Of course, there are other interpretations, unrelated to attention, that could have yielded a similar pattern of thresholds and slopes across conditions. A sufficient increase, under uncertainty, in the variance of some putative “internal noise,” for example, would be expected to produce a similar pattern, both in terms of the thresholds and slopes derived from the *d′_p_* and *d′_s_* data. Another interpretation, again, unrelated to attention, is that, in both the *F* and *R* conditions, observers employed a detection strategy in which the presence of the target was determined *via* a “qualitative change in the character of the masker” (Neff and Green, 1987, pp. 412–413) resulting from the addition of the target. The logic of this interpretation is as follows: in the *F* condition, the same masker sample was presented on all trials within a block. As such, the subjective “quality of the masker” (Neff and Green, 1987, p. 410) was likely relatively stable across trials and thus observers could have simply responded “target present” on those trials on which, due to the addition of the target, the subjective quality of the masker most strongly deviated from the expectation. Randomization of the masker in the *R* condition, by contrast, would have produced large variations in the subjective quality of the masker across trials—regardless of whether the target was added or not—rendering this strategy ineffective; large amounts of modulation IM, therefore, would be expected to occur.

To examine this possibility in more detail, a brief follow-up experiment was conducted. The stimuli (target, set of six maskers, carrier, etc.) and task were the same as in the main experiment but, instead of using a single-interval yes-no procedure, a two-interval, two-alternative forced-choice procedure was used. On each trial, one interval contained the target and one did not; the observer's task was to indicate which interval contained the target. Two observers (two females; 21–22 years of age) who had not taken part in the main experiment participated. Three conditions were tested: (1) a fully-fixed condition, in which the modulation frequency of the masker was the same on all trials and intervals within a block, (2) a between-trial randomization condition, in which the modulation frequency of the masker was selected at random on each trial but was fixed across intervals, and (3) a within-trial randomization condition, in which the modulation frequency of the masker was selected at random on each stimulus presentation. The idea was that a detection strategy based on a qualitative change in the character of the masker would be effective in both the fully-fixed and between-trial randomization conditions due to the stability of the masker quality across trials and/or intervals in these conditions, but would be relatively ineffective in the within-trial randomization condition for the reason noted above. In all three conditions, the modulation depth of the masker was set at –6 dB (as in the main experiment) and the modulation depth of the target was set to the group-mean *F_p_* threshold from the main experiment ( –11 dB) in order to estimate *d′*.^3^

**FIG. 5.**
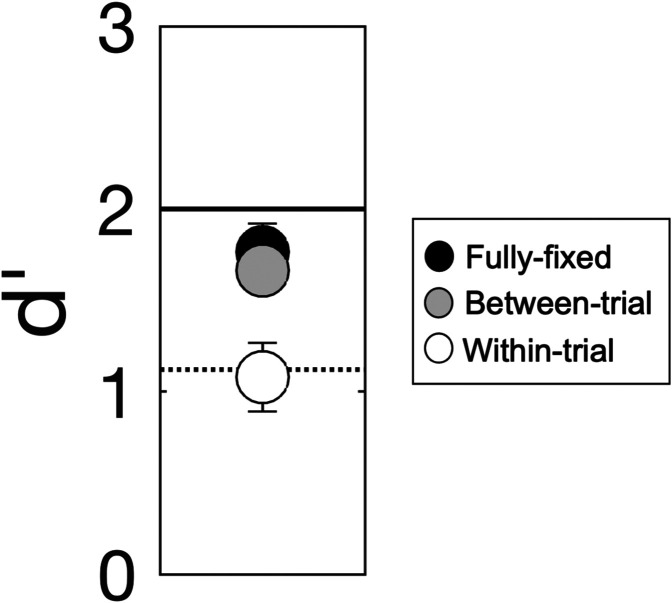
Results of the follow-up experiment. Mean *d′* for the two observers in each of the three conditions: the fully-fixed condition (filled black symbol), the between-trial randomization condition (filled gray symbol), and the within-trial randomization condition (open symbol). Error bars give *±*1 SE of the mean. The horizontal lines show *d′_p_* values for the *F_p_* (solid line) and *R_p_* (dotted line) conditions of the main experiment extracted from the group-mean *d′_p_* psychometric functions at the point at which the target modulation depth ( –11 dB) was the same as in the follow-up experiment.

Figure 5 shows the mean *d′* across the two observers in each of the three conditions tested in the follow-up experiment: the fully-fixed condition (filled black symbol), the between-trial randomization condition (filled gray symbol), and the within-trial randomization condition (open symbol). Error bars give ± 1 SE of the mean. For comparison, the horizontal lines show *d′_p_* values for the *F_p_* (solid line; *d′_p_* = 2) and *R_p_* (dotted line; *d′_p_* = 1.12) conditions of the main experiment, extracted from the group-mean *d′_p_* psychometric functions at the point at which the target modulation depth ( –11 dB) was the same as in the follow-up experiment (cf. Fig. 1).

The first point to be made with respect to the results shown in Fig. 5 is that, consistent with the results of the main experiment both in terms of trend and magnitude, masker-modulation-frequency uncertainty in the within-trial randomization condition (a condition analogous to the *R* condition of the main experiment) had an adverse effect on target modulation detection performance relative to the fully-fixed condition (a condition analogous to the *F* condition of the main experiment): mean *d′* was 1.77 (SE = 0.15) in the fully-fixed condition vs 1.08 (SE = 0.19) in the within-trial randomization condition. Notably, however, there was effectively no difference in *d′* between the fully-fixed and between-trial randomization conditions (the mean *d′* in the between-trial randomization condition was 1.67, SE = 0.03), indicating that fixing the modulation frequency of the masker across intervals of each two-interval forced-choice trial effectively eliminated modulation IM. This is consistent with the notion that detection of the target both here and in the main experiment could have been based on a change in subjective masker quality. The subjective quality judgements could have been based on a variety of cues (e.g., cues related to the temporal structure of the envelope; cf. Strickland and Viemeister, 1996; Viemeister *et al.*, 2005) and thus this finding raises the more general possibility that modulation channels may not be required to explain our results.

The results of the follow-up experiment speak to another possible interpretation of the results of the main experiment, namely, that adaptation of the masker modulation (cf. Tansley and Suffield, 1983; Richards *et al.*, 1997; Wojtczak and Viemeister, 2003) played a role. That is, it is possible that, in the *F* condition, long-term adaptation of the masker modulation resulting from the repeated presentation of the same masker across trials reduced the ability of individual masker samples to produce masking relative to when those same maskers occurred in a random-masker context (i.e., in the *R* condition). If that were the case, however, we would expect to see a difference in *d′* between the fully-fixed and between-trial randomization conditions in the follow-up experiment (i.e., greater adaptation resulting from repeated presentations of the same masker across trials vs only across intervals), whereas none was found (cf. Fig. 5). It is possible, however, that adaptation effects were effectively at their maximum following a single stimulus exposure, in which case no difference in *d′* between the fully-fixed and between-trial randomization conditions would be expected. Indeed, whereas some past studies of modulation adaptation have used relatively long adaptors (i.e., on the order of minutes) in order to achieve adaptation effects (e.g., Tansley and Suffield, 1983; Richards *et al.*, 1997; Wojtczak and Viemeister, 2003), results from modulation forward-masking experiments (e.g., Wojtczak and Viemeister, 2005; Moore *et al.*, 2009; Wojtczak *et al.*, 2011) suggest that modulation adaptation can occur after relatively brief stimulus exposures (i.e., on the order of hundreds of milliseconds), and thus adaptation could still have been a factor in the between-trial randomization condition. Therefore, we cannot rule out the possibility that adaptation to the masker modulation played a role in producing modulation IM in the main experiment.

Finally, we note that off-frequency listening in the modulation domain in the *F* condition may have been a factor in producing the positive threshold differences, and thus modulation IM, in the main experiment. The idea here is that, in the *F* condition, listeners could have attended to a modulation channel tuned to a frequency below the modulation frequency of the target for maskers that were above it (or vice versa) in order to improve the within-channel representation of the target. This strategy would have been difficult to deploy in the *R* condition, however, due to the randomization of the masker across trials, yielding poorer thresholds and modulation IM. While our results are insufficient to determine the extent to which off-frequency listening was a factor, it seems plausible, and therefore must be considered in future work in this area.

## V. SUMMARY AND CONCLUSIONS

The purpose of this study was to determine if masker-modulation-frequency uncertainty in an otherwise typical modulation-masking experiment can have an adverse effect on target modulation detection performance analogous to the adverse effect of masker-frequency uncertainty typically observed in studies of frequency IM. It did, and we took this finding as evidence for modulation IM. Certain aspects of the results lent credence to the hypothesis that modulation IM, by way of analogy to frequency IM, reflects an adverse effect of masker-modulation-frequency uncertainty on the ability to focus selective attention on a target modulation channel. Other aspects, however, were deemed equivocal, and alternative interpretations were considered based on (1) an increase in internal noise under conditions of uncertainty, (2) detection of the target modulation based on a change in subjective masker quality, (3) modulation adaptation, and (4) off-frequency listening in the modulation domain. Each of these interpretations appears plausible in accounting for certain aspects of the results and thus further work is required to fully understand the mechanisms responsible for producing modulation IM.

## Footnotes

^1^ A series of *post hoc* t-tests comparing thresholds among maskers (two-tailed, *p* < 0.05, Bonferroni correction) indicated that the main effect of masker modulation frequency was driven primarily by the elevated thresholds produced by the 16-Hz masker in both the *F_s_* and *R_s_* conditions. Specifically, the group-mean threshold for the 16-Hz masker averaged across the *F_s_* and *R_s_* conditions (−6 dB, SE = 1 dB) was significantly higher than the group-mean threshold (again, averaged across conditions) for the 11-Hz masker (−9 dB, SE = 1 dB), the 91-Hz masker (−11 dB, SE = 1 dB), and the 128-Hz masker (−10 dB, SE = 1 dB). Moreover, the group-mean threshold for the 64-Hz masker (−8 dB, SE = 1 dB) was significantly higher than that for the 91-Hz masker. No other comparisons reached statistical significance at the *p* < 0.05 level.

^2^ In the *R_s_* condition, the 0-dB maximum threshold rule was required in 12 of 48 cases. Seven of those 12 cases were for either the 16-Hz or 64-Hz masker.

^3^ Proportion correct performance was measured for each of the three conditions over six blocks of 50 trials each. The proportion correct score for each block was converted to *d′* (see, e.g., Macmillan and Creelman, 2005, p. 172) without respect to the particular masker sample that was used on each interval/trial (as in the pooled analysis conditions of the main experiment) and the final estimate of *d′* for each observer in each condition was taken as the mean of the *d′* estimates across the six blocks.
